# Supplementary material for: Factors Affecting Antibiotic Prescription among Hospital Physicians in a Low-Antimicrobial-Resistance Country: A Qualitative Study
Source: Antibiotics (Basel). 2022 Jan 13;11(1):98. doi: 10.3390/antibiotics11010098 (PMC8773165; doi:10.3390/antibiotics11010098)
Supplement: Supplementary file 1 [file antibiotics-11-00098-s001.zip › Supplementary Material File S1_Interview_guide.pdf]

Supplementary materials File S1

Full interview guide

1) **What are your thoughts about rational antibiotic prescription?**
2) **What are your thoughts about antimicrobial resistance?**
3) **How do you experience the antibiotic prescription in this hospital, from your perspective?**
   Probes:
- Leads in your department? –is this something you think others experience or are the opinions dispersed?
4) **What influences you when you prescribe antibiotics?**
   Probes:
- Is it factors, persons etc. that influences you?
- Can you think of an antibiotic prescription episode that you remember well?
5) **Can you please tell me about a situation where you had to make a decision about to start, not start or stop antibiotics that you remember in particular?**
   Probe:
- Why do you remember this episode?
- What made you take the final decision?
- Can you look back in you carrier, and recall an episode where you had to make a difficult decision regarding antibiotic prescription?
6) **Do you have any last comments on rational antibiotic prescription?**
